# News about Therapies of Alzheimer’s Disease: Extracellular Vesicles from Stem Cells Exhibit Advantages Compared to Other Treatments

**DOI:** 10.3390/biomedicines10010105

**Published:** 2022-01-05

**Authors:** Jacopo Meldolesi

**Affiliations:** 1 San Raffaele Institute, Vita-Salute San Raffaele University, 20132 Milan, Italy; meldolesi.jacopo@hsr.it; 2 Faculty of Medicine, CNR Institute of Neuroscience, University Milano-Bicocca, 20132 Milan, Italy

**Keywords:** Aβ, APP, BACE-1, clinical trials, exosomes, ectosomes, EVs, markers, miRNA and other non-coding RNAs, MSCs, MSC-EVs, preclinical analysis, PrP^C^ prion protein, tau protein

## Abstract

Upon its discovery, Alzheimer’s, the neurodegenerative disease that affects many millions of patients in the world, remained without an effective therapy. The first drugs, made available near the end of last century, induced some effects, which remained only marginal. More promising effects are now present, induced by two approaches. Blockers of the enzyme BACE-1 induce, in neurons and glial cells, decreased levels of Aβ, the key peptide of the Alzheimer’s disease. If administered at early AD steps, the BACE-1 blockers preclude further development of the disease. However, they have no effect on established, irreversible lesions. The extracellular vesicles secreted by mesenchymal stem cells induce therapy effects analogous, but more convenient, than the effects of their original cells. After their specific fusion to target cells, the action of these vesicles depends on their ensuing release of cargo molecules, such as proteins and many miRNAs, active primarily on the cell cytoplasm. Operationally, these vesicles exhibit numerous advantages: they exclude, by their accurate selection, the heterogeneity of the original cells; exhibit molecular specificity due to their engineering and drug accumulation; and induce effective actions, mediated by variable concentrations of factors and molecules and by activation of signaling cascades. Their strength is reinforced by their combination with various factors and processes. The recent molecular and operations changes, induced especially by the stem cell target cells, result in encouraging and important improvement of the disease. Their further development is expected in the near future.

## 1. About the Alzheimer’s Disease

The present review deals with Alzheimer’s disease (AD), the long-term, neurodegenerative disease that affects the highest number of patients around the world [1,2,3]. For these patients, a severe problem has been, and still is, the lack of fully efficient therapies. After the AD discovery at the beginning of last century, and during the following decades, support to patients could only be provided by their families. The first attempts of drugs, generated by biomedical and pharmacological studies towards the end of the last century, concerned inhibitors of acetyl-cholinesterase and the NMDA receptor. These drugs, however, did not improve significantly the AD state, but induced only moderate slow-down of its development [3,4].

During the following years, additional attempts of drug production and employment have been made, however with little success. The promises of the last decade, concerning two main developments, the blockers of β-secretase (the enzyme, also known as BACE-1, that governs the generation of the key factor of the disease, the amyloid peptides, Aβ-42 and Aβ-40 [5,6]) and the concomitant immunotherapies of the phosphorylated protein tau [7], are intensely investigated but not yet commonly employed [8]. Only recently aducamumab, a new drug of limited relevance, has been approved in the U.S. for AD treatment. Concomitantly, antibodies targeted to AD peptides and proteins are in advanced stages of clinical trials [9]. We conclude that, although slowly, some traditional anti-AD approaches, concerning a few drugs and antibodies, are reaching advanced stages of evaluation [9]. Such progress, however, does not cover the entire panel of drugs needed in the area. The discovery, during the last decade, of brain extracellular vesicles (EVs) has opened perspectives to new therapy of diseases, including AD, that are now seriously considered.

EVs, secreted by all cells, include two types of vesicles, exosomes and ectosomes, different in many properties including size (average diameters of about 70 and 250 nm) and mechanism of release from their cells of origin (exocytosis of endocytic vacuoles, the multi-vesicular-bodies [MVBs], containing many exosomes, and shedding of ectosomes assembled at plasma membrane rafts). Updated details about these two types of vesicles and their functions can be found in [10]. However, exosomes and ectosomes, sharing important molecular properties and effects, are considered together as EVs secreted by mesenchymal stem cells (MSCs). Due to their origin, these vesicles are called MSC-EVs. By their navigation and specific fusion followed by release of their cargo proteins and miRNAs, the MSC-EVs have been shown to induce novel forms of cell-free therapy [11]. Various therapeutic effects of MSC-EVs have been found to be appropriate for AD [3,8,12].

The present review has been planned in Section 4. As a necessary beginning, I have summarized the established knowledge about the AD (Section 1), MSCs, and MSC-EVs (Section 2), including important aspects appeared recently. The following Sections deal with two main pathways of AD therapy, still in continuous development (Section 3 and Section 4). The first, including BACE-1 and antibodies, illustrates mostly technical developments; the second, dealing with MSC-EVs, illustrates many new properties that have appeared during the last year. The present state of AD therapy is also relevant for its development expected in the near future.

For many decades, AD therapy remained absent or marginal. Beginning a few decades ago, a coordinate amyloid hypothesis of the disease was presented, then was discussed, and, finally, generally accepted [13,14]. In parallel to such interpretation, novel therapeutic strategies were developed, contributing to the understanding of the disease [4,5,8]. The presentation of the present Section is organized in four subsections. The first three (Section 1.1, Section 1.2 and Section 1.3) deal with the molecules and mechanisms that govern the properties and the development of the disease. The fourth Section 1.4 does not deal with AD directly, but with its markers, the tools by which the disease, its components, and functions are distinguished from the properties of other diseases.

### 1.1. Role of β-Amyloid Peptides and Their Co-Operators

**Figure 1:**
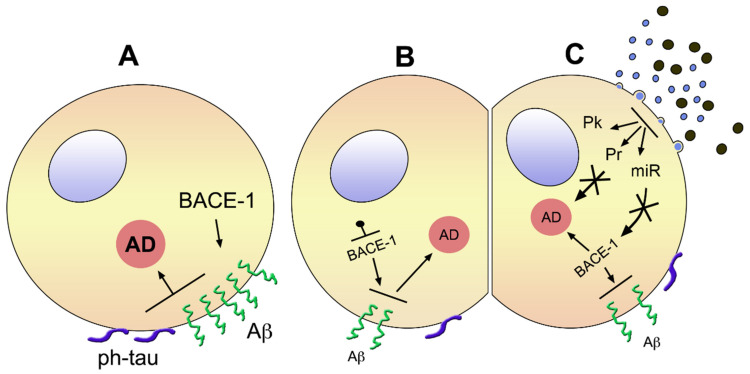
Brain cells with ongoing early AD before (**A**) and upon treatment with a blocker of BACE1 (**B**) or MSC-EVs (**C**). (**A**) shows a cell apparently in good state (early AD), affected by a high level of BACE-1 inducing high concentration of Aβ accompanied by surface phosphorylation of tau, i.e., pathological properties of the two factors responsible for the severity of the disease, which is shown in the large orange circle. (**B**) shows a cell similar to the one in (**A**), treated, however, by a blocker of BACE-1. The smaller size of the enzyme name means its decreased activity, with a lower surface density of Aβ and phosphorylated tau. Consequently, the state of AD, revealed by the small size of its orange circle, is reduced. The cell in (**C**) is similar to the one in (**B**), however, its therapy is different. The EVs shown on the top-right are a natural mixture of small exosomes and larger ectosomes. Their fusion to the cell shown at the cell surface near the EVs induces in the cytoplasm generation of protein kinases (Pk), proteins (Pr), and miRNAs, which induce apparent inhibition of AD (small orange circle), generated directly or via inhibition of BACE-1 (small size) and other effects. Not shown is that the effects of EVs can be induced by vesicles of a single type, isolated or produced, engineered or not, and administered to the patient by intravenous, intracerebral, or intranasal injection.

Only a very small fraction of ADs, the familial form, originates from gene mutations (10% of amyloid precursor protein, APP; 90% of presenilins) that induce effects analogous to those of the other, non-familial forms [15]. (Figure 1). The initial, critical step of the latter forms, around 99% of the disease, starts with proteolysis of APP and generation of the amyloid peptides Aβ-42 and Aβ-40 (together called Aβs), induced in sequence by two enzymes, presenilin 1 and presenilin 2. In order to initiate its signaling cascade, Aβ needs to bind as a sort of receptor linked to the plasma membrane, a prion protein (PrP^c^), ubiquitous to mammalian neurons [16]. Upon its interaction with Aβ, PrP^C^ establishes another specific binding to mGluR5, a trans-membrane metabotropic glutamate receptor which governs part of the effects induced by Aβ [17,18]. Mechanistically, mGluR5 mediates the functions of its complex with Aβ-PrP^C^ by interacting with several post-synaptic proteins, including the Go protein Homer b/c and various kinases, the Ca^2+^-calmodulin-dependent protein kinase II, the c-Jun terminal kinase, and various tyrosine protein kinases. Such complexes modify the phosphorylation state of various cell proteins. Moreover, mGluR5 has been shown to govern the list of neuron-glia interactions, a process of critical importance in the brain [18,19].

### 1.2. Tau Inside and Outside Neurons

Tau is a microtubule-associated protein abundant in the neuronal cell cytoplasm. In AD and other neurodegenerative diseases, tau over-expression is followed by its aggregation, misfolding, hyper-phosphorylation, and gain of toxic functions [20]. All these properties are spread via EV traffic, from the affected cells to other neurons and microglia [20,21]. Following accumulation of high and low molecular weight forms of tau, the cells start inducing its detachment from microtubules with relocation from axons to somato-dendrites, followed by loss of dendritic spines, disruption of synaptic plasticity, and alteration of mitochondria [20,22,23].

Upon interaction with proteins at the inner leaflet of the plasma membrane, tau undergoes translocation to the cell surface (Figure 1A) [24]. By extracellular cooperation with Aβ, mediated by PrP^C^, tau affects synergistic effects in various brain areas [20,24,25], including integrity of multiple neuronal circuits, with ensuing defects in learning and memory [26]. Pathology of tau combined to Aβ contributes to a large fraction, but possibly not all, cognitive impairments induced in AD.

### 1.3. A Few Additional Factors of AD: Lipoprotein apoE4, Insulin Receptor, Astroglia and Microglia

Here, I intend to summarize the role of some of the factors known to reinforce the pathological effects of Aβ and tau in AD. Expression of apoE4 lipoprotein was first identified as a risk factor for late-onset ADs. Its expression has been shown to strengthen the activation of Aβ and the hyper-phosphorylation of tau [27], with increased severity of neurodegeneration [28]. Involvement was shown also for NMDA and insulin receptors. A role of the latter was first suggested by the more frequent appearance of AD in patients affected by type-2 diabetes and obesity. Resistance of the brain insulin receptor induced by Aβ and tau induces increased levels of the first, and increased phosphorylation of the second [29]. Finally, both astrocytes and microglia, in balance with neurons, have been shown to play critical roles in AD. Moreover, such glial cells are involved in the regulation of processes of relevance in AD, i.e., neuroinflammation and immune responses [30,31].

### 1.4. AD Markers

Progress about AD has often been due to recognition of properties that had remained unknown or under-considered for long time. Recently, some of such properties, identified for studies in specific MSCs, have become more and more relevant. The tools competent for such answers are called markers, or biomarkers in case they are of biological origin. As examples, I mention the identification and function of genes; the expression and distribution of cell components; functional defects and mutations of lipids, receptors, and transporters; and the age of patients and their aging. During the last years, biomarkers have been integrated with markers of various origin. Best results for important tasks (e.g., distinction of AD from other diseases; state of AD; identification of AD subgroups; and many others) have been obtained by the co-use of multiple types of markers [32,33].

## 2. General Properties and Functions of Mesenchymal Stem Cells and Their Extracellular Vesicles

Stem cells, identified many decades ago, had been first considered specific of early development. Around 1970, some of their specific effects, including self-replication and multidirectional differentiation, started to emerge. At the beginning of 2000, new effects of MSCs appeared, and interest about the role of these cells started to grow. Therapies by MSCs, inducing tissue repair and lesion regeneration, were first demonstrated in bones and cartilages [34]. Soon thereafter, therapies of specific diseases started to appear, first in the same organs, then in many others, including the brain and its diseases [12]. Interestingly, the results obtained by basic studies were often considered extensible to clinical medicine [35,36]. Concomitantly, MSC treatments were shown to induce various forms of immunology together with attenuations of inflammatory processes [37]. Initially all such effects were thought to be due to paracrine fusions of MSCs to their target cells, reinforced by bioactive factors, such as cytokines and growth factors, as well as by their MSC-EVs.

The MSC therapy studies, initially carried out in mice, were found to induce mostly positive results. Their extension to human diseases initially confirmed the positive results, with expected development of clinical trials [35,36]. Shortly thereafter, however, positive results were followed by the appearance of dangerous risks, including immune rejections and various types of cancer [38]. Such unexpected inconveniences lead to rapid reduction and then repulsion of the MSC therapies, accompanied by withdrawal of initially proposed clinical trials. Recognition of serious problems in MSC therapies opened the way to corresponding studies about their secreted vesicles [12,39]. Such studies demonstrated recapitulations by MSC-EVs of many therapeutic effects of MSCs. In addition, such recapitulations were accompanied by unexpected improvements, including increased safety and faster tissue penetrations. In addition, the inability of MSC-EVs to self-replicate was shown to prevent various risks typical of MSCs. Effects, such as activation of the immune system and prolongation of the therapeutic effects, confirmed the MSC-EV therapy to be favorable when compared to the cell-based approaches [39,40].

As already mentioned, members of the MSC families include moderate protein differences in the endosomal cisternae and plasma membrane rafts, the two pathways of EV generation. Thus, critical properties of multiple MSC-EVs also exhibit some heterogeneity. It should be emphasized, however, that the MSC-EVs employed for investigation and therapies do not come from the stroma of any tissues, but only from four of them: bone marrow, adipose tissue, umbilical cord, and blood from that cord, chosen because of their accessibility. The heterogeneity among these MSC-EVs concerns their proteins and, especially, their miRNAs. The vesicles most active against prolonged brain diseases, including AD and multiple sclerosis, are those from MSCs of adipose tissue origin. Vesicles from the umbilical cord affect, especially, the acute diseases and operate in damage repair; those from bone marrow are especially active in tissue regeneration [41,42,43]. Knowledge about the role of MSC-EVs on various diseases, confirmed by preclinical analyses, stimulates their use as therapeutic tools.

## 3. Role of the Extracellular Vesicles from Mesenchymal Stem Cells: Recent Views about the Alzheimer’s Disease

At present, the brain MSC-EVs, secreted by both neuronal and glial stroma cells, are recognized as versatile vesicles of communication, operative via molecules such as proteins, RNAs, lipids, and metabolites, involved in many processes: synaptic plasticity, nutritional metabolic support, nerve growth and regeneration, and mitigation of inflammatory processes. The properties of their cargoes and their membranes are largely known [44,45,46]. In various studies these vesicles have been shown to protect neurons from pathological processes; for example, from ischemic conditions [47]. In the hypothalamus, stem cell EVs control aging through some their miRNAs [48].

### 3.1. New Markers

Before covering the mechanism of the MSC-EV action and their operative effects in AD, I intend to illustrate the role of their new markers, various types of molecules already introduced in the Section 1.4 [32,33]. In view of their unique properties to distinguish among analogous diseases, the present interest about markers is considerable. Many previous results, including the presence within MSC-EV cargo of typical AD proteins, such as Aβ and highly phosphorylated tau, have been recently confirmed [49]. Other cargo components, i.e., their miRNAs, have also been intensely investigated [50,51]. Additional non-coding cargo components, including lncRNAs, circRNAs, snoRNAs, tRNAs, and piRNAs, are also widely employed as markers. Good examples of these RNAs have been identified and employed for the characterization of ADs [52,53]. Another study has been focused on SNORDs (Box H/ACA small nuclear RNAs) transcribed from imprinted genomic loci. Some of these non-coding RNAs have been shown to accumulate within MSC-EVs [54]. Interestingly, as reported by previous [32] and recent [54,55,56] studies, the preliminary and advanced stages of AD can be easily distinguished. The age of AD patients is critically important for their therapy, as specifically discussed in Section 4.

EVs released from MSCs fuse with neurons and glial cells of the brain and spinal cord, acting primarily by the proteins and miRNAs of their cargoes. Such EV presence can influence various processes of neural tissues, including synaptic plasticity, metabolism and nerves, and reducing effects of inflammation and toxins, as well as pathologies such as those induced by traumas and ischemias. The effects summarized here, present already in normal conditions and in localized pathologies, participate in the therapy of MSC-EVs in AD, integrating these effects to many others, including those of Figure 1C.

### 3.2. Actions of Extracellular Vesicles on Neural and Glial Cells, Recovery from Traumas and Immune Reactions

MSC-EV fusions have been shown to play critical roles in the intercommunications among brain cells, occurring during health and in various pathologies (Table 1). Numerous protective results have been reported, including development of synaptic plasticity, nutritional metabolic support, nerve regeneration, inflammatory response, and elimination of toxic components [46,57]. Traumas and ischemic lesions can be induced in both the brain and spinal cord. In many cases, the recovery induced by low doses of MSC-EVs has been shown to start with activation of few receptors, followed by phosphorylation of kinases and various factors, and by activation of miRNAs [56,57,58,59]. Analogously, MSC-EVs have been shown to mitigate trained immune responses induced by innate cells, with distinct local repair [60]. In addition to the effects on neurons, MSC-EVs modulate their responses and suppress aggressive effects induced by glial cells, both astrocytes and microglia [61,62].

## 4. Present Therapies for Alzheimer’s Disease

As already emphasized in the introduction, the first pharmacological therapies of AD, due to the inhibition of neuronal signaling, remained only marginal. A few years later the interest moved to blockers of the enzyme β-secretase (in pharmacological studies reported here almost always called BACE-1), necessary for the generation of the critical Aβ peptides (Figure 1B), accompanied by another approach, the immunotherapy of both tau and Aβ. Rapidly, the enthusiasm about BACE-1 grew considerably. However, intense studies of many AD patients came out largely unsuccessful, as documented by clinical trial attempts that were either concluded without benefit or discontinued [4,5]. Clear positive effects were observed only in healthy persons destined to develop the disease in the future, and in very early patients [3,63], identified by appropriate markers [33,54,55,56]. Based on the long-term experience in the field, the investigation of BACE-1 and immune therapies has been pursued up to now. Their present developments are illustrated in Section 4.1.

The other tools relevant to AD, the MSCs and, especially, their secreted extracellular vesicles MSC-EVs, have been introduced in Section 3. Some established properties of these cells and vesicles are reported there. Due to these properties, MSC-EVs have a role in the therapy of several diseases, shown in previous publications see [11,38,64], including the AD (Figure 1C) [3]. The present state of such therapy is discussed critically in Section 4.2 and Section 4.3.

### 4.1. BACE-1 Blockers and Anti-Tau, Anti-Aβ Antibodies

The present approach to BACE-1 blocker studies is based on the identification of hundreds, or even thousands, of such possible drugs analyzed by multiple distinct procedures. In [65], five drugs are classified as high score. The employed approach appears promising for the identification, in the future, of appropriate new BACE-1 inhibitors. At present, such drugs are still questionable for their incomplete effects and their associated risks. In a concomitant study [66], a single molecule, 11-oxotigogenin, was found to be the best, high affinity BACE-1 inhibitor investigated, which, however, is still warranting validation. In addition to its effects, 11-oxotigogenin can pave the way for designing chemical scaffolds, another approach to discover potent BACE-1 inhibitors. Additional articles suggest new ways for the development of future clinical trials about BACE-1 inhibitors. In particular, computational modeling methods are emphasized to promote profound applications in drug discovery strategy [67,68]. A property of BACE-1 blockers confirmed by preliminary results is that their problems are due to the occurrence of adverse effects. Administration of low levels of appropriate BACE-1 blockers has been shown to avoid adverse effects while achieving some efficacy for AD prevention [68]. At present, other tools investigated for AD protection are metalloproteases active on APP metabolism [69].

As far as the anti-Aβ and anti-tau antibodies, no therapeutic strategy is potent enough to block the progress of the disease. However, immunotherapies against Aβ and tau proteins remain popular for AD therapy. The best anti-AD results have been obtained by targeting the mid-region of the extracellular form of tau. Antibodies that target these proteins are in advanced stages of clinical trial [9]. However, fully effective therapeutic strategies remain to be developed [70].

### 4.2. Extracellular Vesicles from Mesenchymal Stem Cells. Results in Mice

There is no need to emphasize that the results about AD therapy have been extensive in mice, but most important in humans. Studies of MSC-EVs are established in mice models of the disease, such as APP/PSI and 3xTg, which are easy to employ. An important aim of mice model studies has been the establishment of preliminary experience, preliminary to subsequent studies in humans.

The well-known therapy, induced in various types of cells by MSC-EVs, has also been demonstrated in the brain, where hippocampal neurogenesis was confirmed during the AD disease. MSCs cells, in fact, hold an immense potential to regulate and protect neurogenesis [71,72]. The main mechanisms of such effects are the fusions of secreted MSC-EVs, with ensuing release to their target cell cytoplasm, important components of their cargo, especially nucleic acids, together with other tools for therapy. Among the effects important for AD are the repression of BACE-1 and Aβ levels with increase in sphingosine-1-phosphate, all events induced by of MSC-EVs protective of neurons against AD [73,74,75]. An important aspect to remember is that, due to their variable composition and/or deregulation and distribution, some MSC-EVs do not reduce, but contribute to the severity of the disease [76]. When using experimental EVs, therefore, their actions need to be preliminarily established.

Another operational property by MSC-EVs deals with their administration. In general, the EVs penetrate through the blood–brain barrier. Therefore, their simple tail-vein injection induces efficient effects to the mouse brain, specifically improving its cognitive impairment, with reduction of Aβ aggregation and neuronal loss. Effects restored by MSC-EVs include [Ca^2+^] oscillations, dendritic spine increases, action potentials, and mitochondrial reactivations [77]. Analogous effects are induced by MSC-EVs administrations closer to the brain, for example, by intra-cerebral injection or intra-nasal delivery [78,79]. In mice, the therapeutic effects are particularly effective at early stages of the disease, when the fully established symptoms are still limited [54,78,79,80]. Interestingly, the therapeutic effects of MSC-EVs do not depend only on the whole vesicles. Rather, their released cargo miRNAs induce inhibition of BACE-1 expression and activation of intracellular signaling cascades, such as those operative along the Wnt/β-catenin and the AKT/GSK-3β/β-catenin pathways [81,82]. Therefore, the mechanisms of MSC-EV action appear more complex than previously proposed. Moreover, EVs activate not only neurons, but also astrocytes and microglia, with ensuing reduction of neuroinflammation [61,83].

### 4.3. Extracellular Vesicles from Mesenchymal Stem Cells. Results in Humans

Compared to mice, a lower number of AD studies have been carried out in humans. However, the available knowledge is relevant. Recent results have confirmed the strong potential for MSC-EV as biomarkers for and therapeutic agents against AD [83,84,85]. Symptoms observed, including restoration of homeostatic levels, protection of synapses, and improved cognition [86], are analogous of those of mice. In fact, AD and a number of other diseases are protected by MSC-AVs from neurodegeneration, with positive effects on tissue repair and neural regeneration [87].

As reported in Section 4.2, dealing with mouse therapy [54,78,80], various human therapy studies have been carried out by preclinical analyses. To be fully comprehensive, however, the results obtained should be analyzed by clinical trials [83,87,88]. Due to the paucity of standard protocols and mechanisms available for the procedures, the development of MSC-EV-dependent therapies has been challenged [89,90]. It should be mentioned, however, that some such properties, in particular the drug accumulation within MSC-EVs, have been approved for specific targeting according to recent technical advances [91,92]. Therefore, clinical trials are expected to become common in the near future.

A very important concept dealing with human MSC-EVs therapies is based on vesicle engineering developed from conditioned media [93,94]. In these cases, the engineering depends on the good manufacturing practice (GMP), relevant in the selection of materials, the manufacturing, and the quality assays employed. The cells/vesicles are engineered by insertion of changes, both at the surface and within the lumen, and by assuring preloading of drugs followed by binding and fusion to appropriate target cells, with discharge of drugs within their cytoplasm [93,94,95]. Despite the efforts made during the last decades, the use of these approaches to diseases has been limited. Based on the variability of the results, the employment of engineering needs to be moderate. In contrast, results with MSCs and, even better, with MSC-EVs, have been much more successful due to the combination of engineered and natural approaches, efficient especially for regenerative medicine. The ensuing MSC-EVs will be competent for cell fusion and cargo diffusion, two typical processes by which key cargo molecules flow to target cells [96,97,98].

## 5. Conclusions

AD, a medical process that for many decades has remained marginal, with no serious therapeutic chance for many millions of patients, started to emerge at the beginning of the present century, when the increased knowledge about the molecular mechanisms governing initiation and severity of the disease have been finally established. Unfortunately, however, the efforts made by pharmacologists and other scientists, working independently and within industries, have remained frustrating for several years. In fact, the efforts to reduce Aβ synthesis by blockers of BACE-1, and those with anti-tau and anti-Aβ antibodies, were found unable to reduce the structural devastations induced by the disease in critical areas of the brain. A posteriori, it is not surprising that the drugs, inactive on fully developed disease patients, are effective in healthy persons destined to develop the disease in the future, and in patients at an early stage of the disease, reducing and slowing down their later development of AD [33,54,55,56].

Knowledge about AD therapy by MSC-EVs has been initially developed based on the therapeutic role first recognized to MSCs, including those of the brain, and then transferred to their secreted vesicles, with disappearance of the associated adverse effects and presence of advantages. Despite these properties, the interest about MSC-EV therapy grew only slowly, stronger in science than in medical practice. Such difference with respect to blockers of BACE-1 appears now unreasonable. In fact, the two systems, despite their distinct mechanisms of action, are not fully independent. MSC-EVs induce a decrease in BACE-1 and Aβ. Moreover, both BACE-1 blockers and MSC-EVs induce increases in factors, such as sphingosine-1-phosphate, that co-mediate their effects on AD [73,74]. In conclusion, the relevance of the results obtained by MSC-EVs is highly significant. An independent advantage is their engineering at the surface and within the lumen, assuring preloading of drugs followed by binding and fusion to appropriate target cells [93,94,95].

The comparison of the two therapeutic approaches, by BACE-1 blockers and MSC-EVs, yielded interesting results. At present, most convincing data have been obtained by the second approach, defined the “new frontier for regenerative medicine” [96,97,98], especially when its results were integrated at a more favorable level than those of other agents and mechanisms, for example, [99]. Combinations of this type had been previously hypothesized for BACE-1, however, a few years ago [100]. At present, the MSC-EVs in various properties [83,84,85,86,87,88,89,90], in drug delivery [91,92], and in combined employment through a miRNA-BACE-1 axis and various pathways addressed to β-catenin [81,82], have yielded very encouraging results. The new findings concerning MSC-EVs and their therapy agents, considered also in their possible combinations as mentioned here, confirm the improvement of AD therapy, with substantial advantage for the whole population of patients, existing already now and with improvement expected in the next few years.

## Figures and Tables

**Table 1:** Effects induced by fusion of MSC-EVs to neurons and glial cells and tissues.

Areas	Agents	Processes	Pathologies
		synaptic plast	inflammations
brain	proteins	metabolism	toxins
spinal cord	miRNAs	nerve growth	traumas
			ischemias

## Data Availability

The data of this review will be available to all scientists of scientific interest.
